# Supplementary material for: Microbial Diversity Dynamics in a Methanogenic-Sulfidogenic UASB Reactor
Source: Int J Environ Res Public Health. 2021 Feb 1;18(3):1305. doi: 10.3390/ijerph18031305 (PMC7908407; doi:10.3390/ijerph18031305)
Supplement: Supplementary file 1 [file ijerph-18-01305-s001.pdf]

SUPPLEMENTARY INFORMATION

Microbial diversity dynamics in a methanogenic-sulfidogenic UASB reactor

E. Fernández-Palacios, X. Zhou, M. Mora, D. Gabriel*

GENOCOV Research Group, Department of Chemical, Biological and Environmental Engineering, Escola d'enginyeria, Universitat Autònoma de Barcelona, 08193 Bellaterra, Spain (*corresponding author: David.Gabriel@uab.cat)

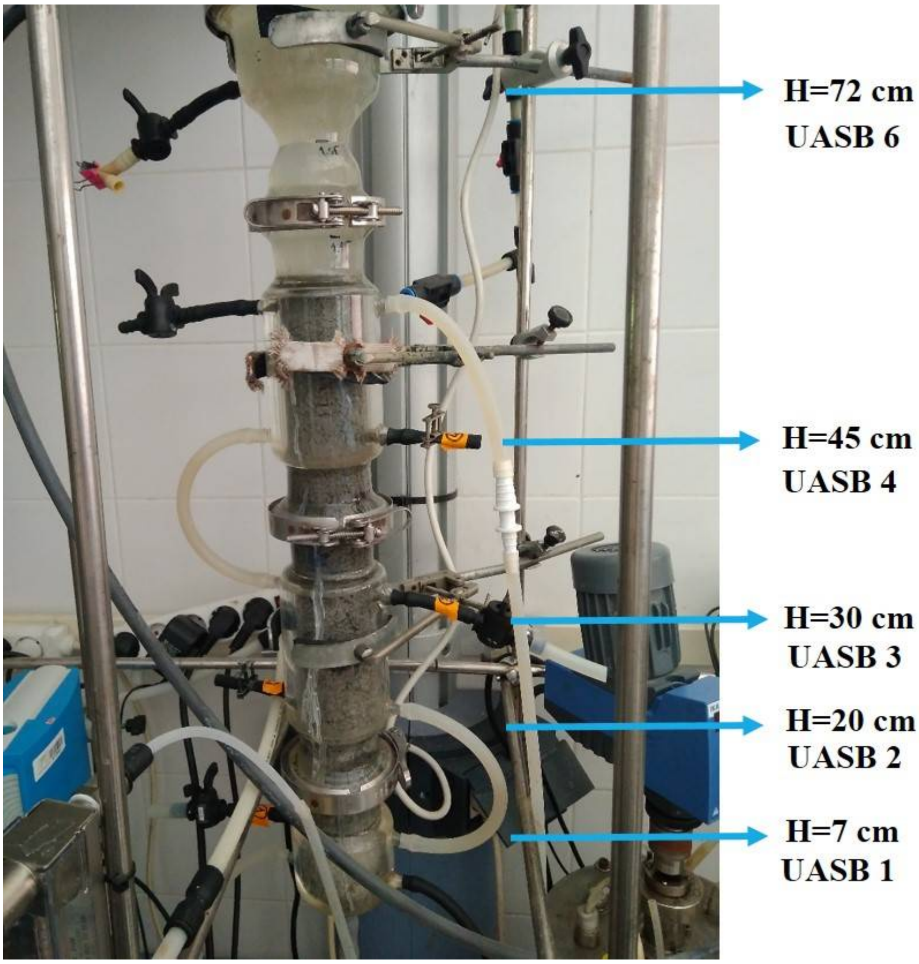

Figure S1. UASB reactor with sampling points at different heights (H).

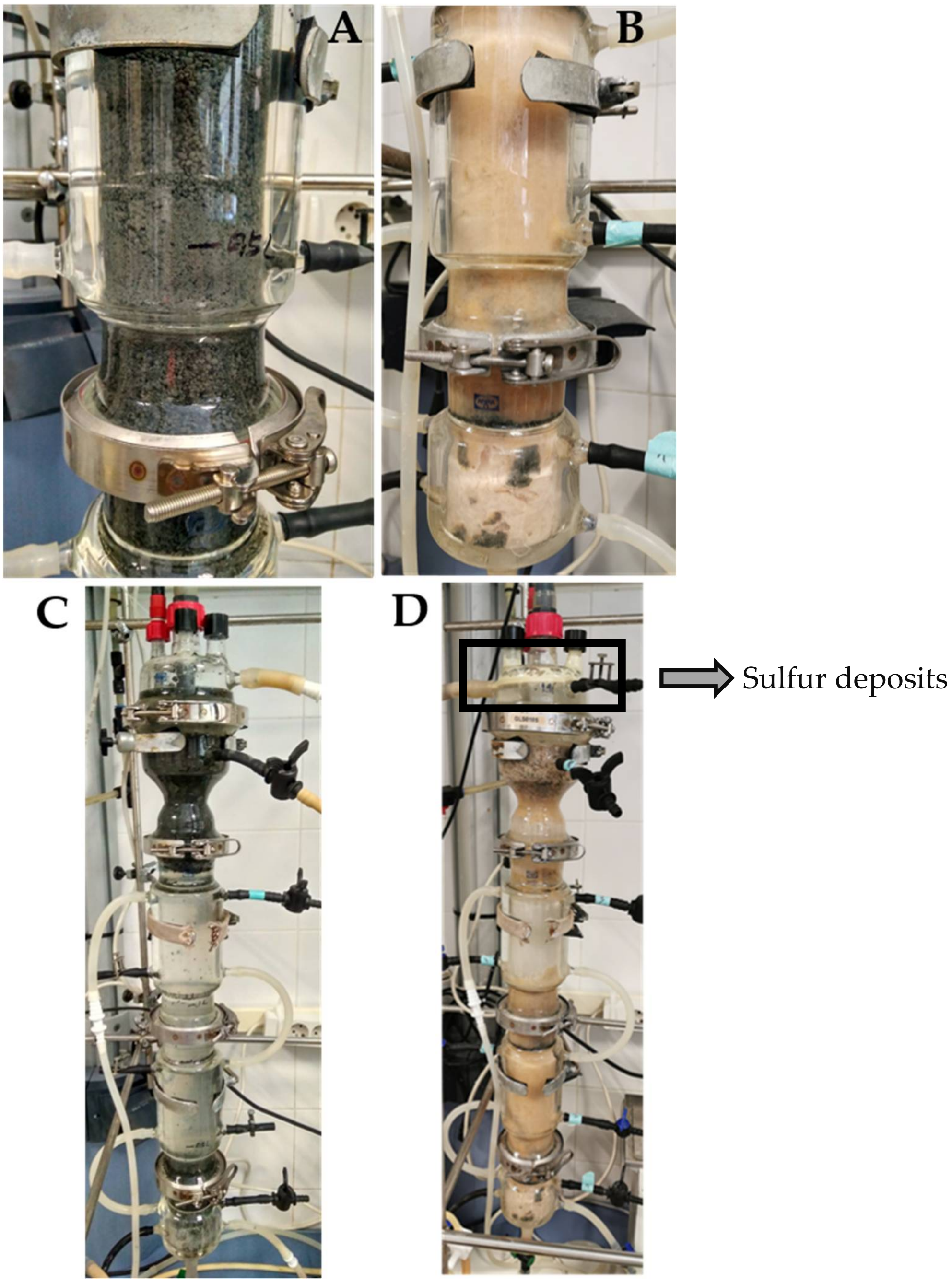

Figure S2. UASB reactor along the long-term operation. A and C) Granular sludge the day the reactor was inoculated; B and D) Slime attached to the reactor the last day of the operation (day 550).

Table S1. Physical-chemical analysis of crude glycerol.

**Parameters analyzed**	**Glycerol**
Organic Material	34.0 %
Water	56.0 %
Soluble salts	5.0 %
Elemental sulfur	2.3 %
COD	500 mg O_2 L^{-1}
BOD_5	345 mg O_2 L^{-1}
Total solids	374 g kg^{-1}
Volatile solids	295 g kg^{-1}
Kjeldahl nitrogen	5100 mg L^{-1}
pH	5.9

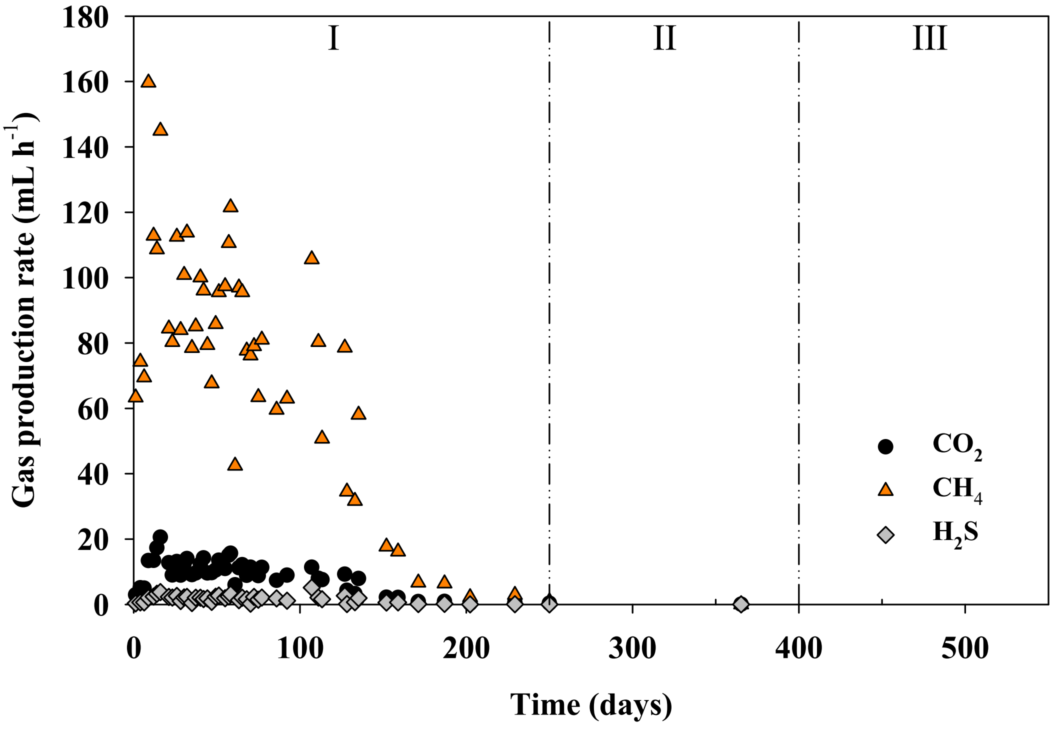

Figure S3. Gas production in the UASB reactor along the long-term operation.

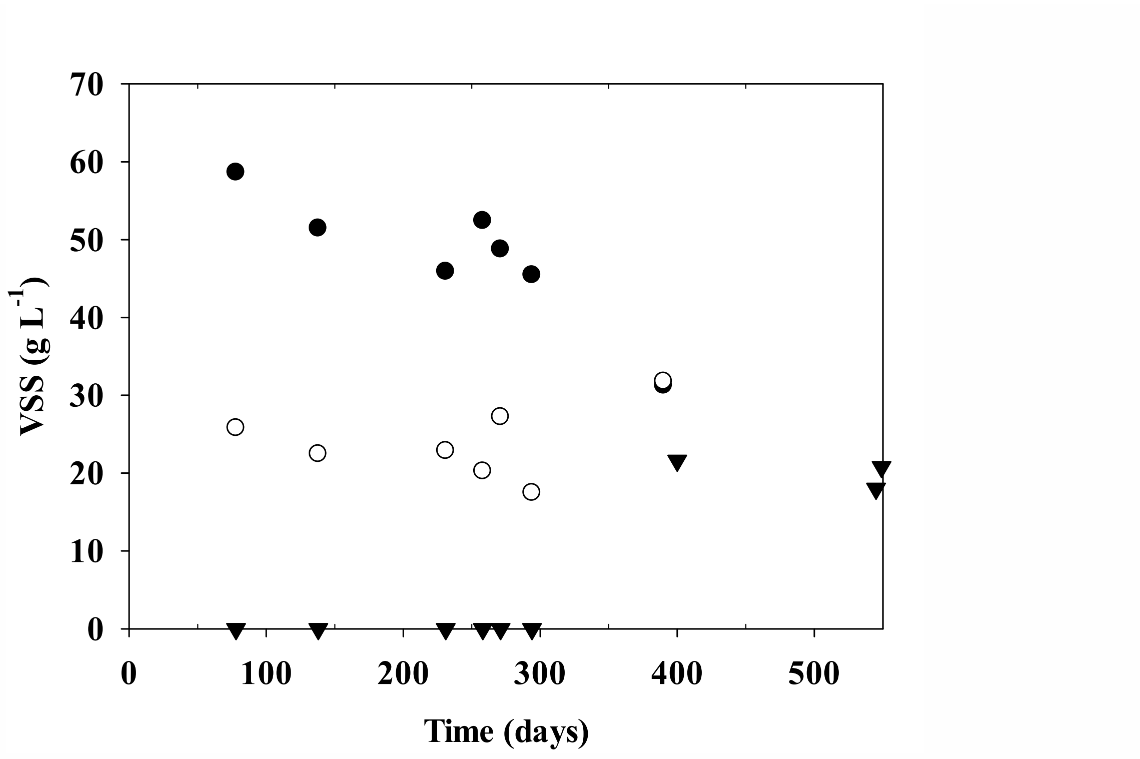

Figure S4. Concentration of Volatile Suspended Solids (VSS) measured on UASB 1 (●), UASB 3 (○), and UASB 6 (▼).

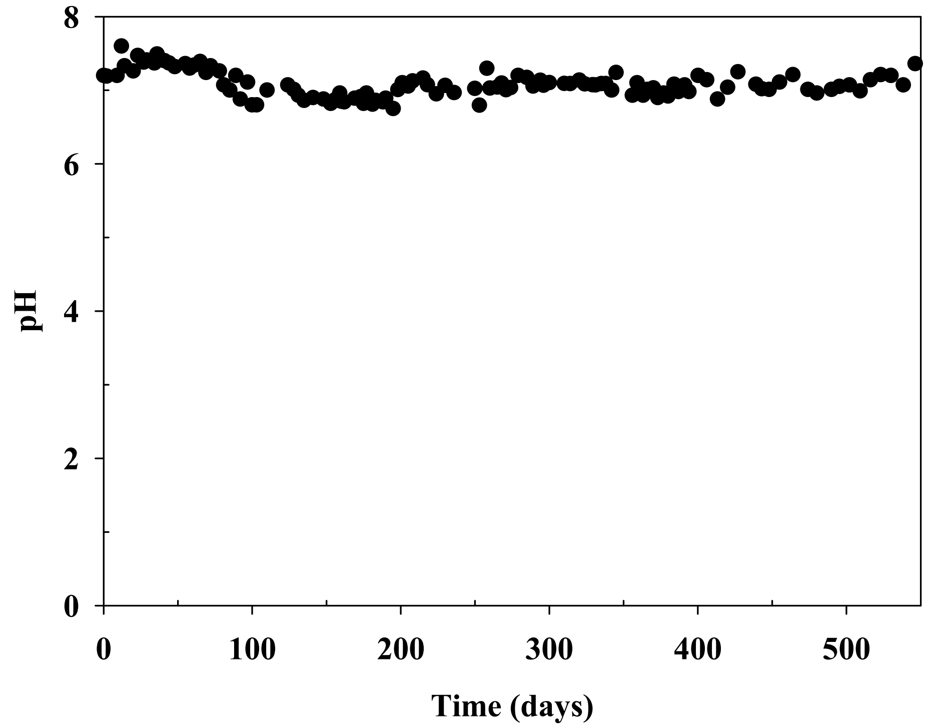

Figure S5. pH values measured in the outlet of the UASB reactor along the long-term operation.
